# Eye injuries: improving our practice

**Published:** 2015

**Authors:** Daksha Patel

**Affiliations:** E-learning Director: International Centre for Eye Health, London School of Hygiene and Tropical Medicine, London, UK.

**Figure:**
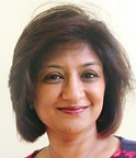
Daksha Patel

Most eye health workers are involved in managing trauma. In fact, ocular injuries around the world make up a major part of daily ophthalmic clinical practice. Eye injuries range from mild, non sight-threatening, to extremely serious with blinding consequences.

## Epidemiology

Epidemiological data on ocular trauma is limited. A review undertaken for the World Health Organization (WHO) in 1998^1^ estimated that injuries were responsible for the following:

- 1.6 million people blind in both eyes
- 2.3 million people with low vision in both eyes
- 19 million people blind in one eye
- 55 million people with eye injuries that resulted in restricted activities for more than one day a year.

The demographic pattern (age/gender) of ocular injuries varies with the environment and cause of injury. The general pattern is that of a curve with two peaks: one in the age group 5–25 years and another in people aged 70 years and over. Compared to women, the risk of eye injuries in men is four times higher.

Accurate data – essential for guiding management and prevention – has been difficult to record or compare, due to a number of factors.

- The different environments in which injuries occur
- The wide range of causes
- The wide spectrum of clinical (anatomical) presentations
- Different data sources, e.g. hospital discharge data, out-patient visits
- The lack of a widely used standardised template for reporting injuries.

**Figure:**
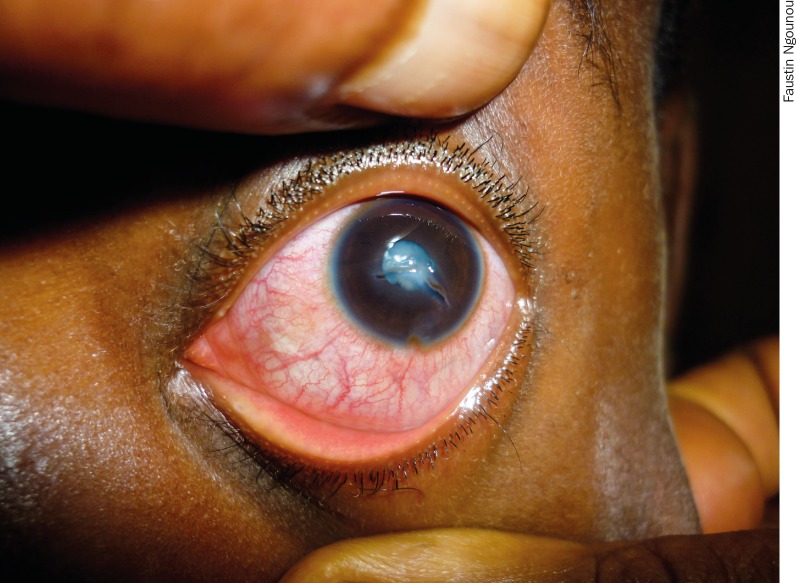
A 10-year-old with corneal laceration and traumatic cataract after a stone hit him in the eye. CAMEROON

**ABOUT THE ISSUE**

This issue of the *Community Eye Health Journal* is about eye injuries, including approaches for prevention and tips on how to assess, classify and manage them. Eye injuries affect people, not just eyes. People with eye injuries are in pain and have been through what was very likely a terrifying experience for them; they will also be anxious about their vision. We explain how to reassure and support patients, despite the difficult circumstances in which they find themselves. We also introduce the Ocular Trauma Score (OTS), based on the BETTS classification, which is there to help clinicians estimate the visual prognosis of an eye injury and guide referrals. It is particularly helpful when talking to patients and their family members about what to expect. The OTS isn't perfect, however – it is correct 4 times out of 5 which means that clinicians must always apply their best clinical judgement when using it. Also, the OTS is only valid if the eye injury has been managed correctly. We hope that our article on the management of injuries will provide useful reminders. Enjoy the issue!

## Assessment and the BETTS Classification

The introduction of the Birmingham Eye Trauma Terminology System (BETTS)^2^ in early 2000 provided a standardised and simple system to describe mechanical injuries to the eye globe. The panel on page 43 provides an outline of this classification, which is applicable to clinical practice and can also be used to audit and create an appropriate registry for injuries. In this issue we look at how BETTS is used to guide the clinician in management.

In all eye trauma cases, the main concern of patients and their families is the visual prognosis. To address this, the Ocular Trauma Score (OTS) has been developed; it is based on the BETTS classification system and is used to calculate prognosis (with the assumption that the trauma is managed optimally). On page 44 we introduce the OTS and demonstrate how it may be used.

## Prevention and management

In general, it seems that people assume that eye injuries are the result of ‘accidents’, i.e. that they are outside of human control. It is not always the case – eye injuries are often preventable. This assumption might go some way towards explaining why, in many countries, not much attention has been given to the development of strategies for eye injury prevention.

**‘The first step in prevention is to understand the local causes of ocular injuries and their patterns’**

The first step in prevention is to understand the local causes of eye injuries, and their patterns. This is why it is important to establish a local injuries register that uses the BETTS classification system and includes age, gender, place and cause of injury. This evidence can guide the development of local prevention interventions, such as protective eyewear in the workplace, legislation and enforcement about the use of seat belts, and first aid management of agricultural eye trauma. Data will also be comparable with other regions and other countries.

In many low- and middle-income countries, trauma cases are often complicated by late presentation and/or previous inappropriate intervention. To have a well-trained first contact person at the primary level is therefore critical for the correct assessment and management of an eye injury.

## Conclusion

From a public health perspective, neither bilateral nor unilateral blindness data provide a complete picture of the impact of ocular trauma on society. Severe ocular trauma requires expensive hospitalisation and specialist treatment, and often prolonged follow-up and visual rehabilitation. This has significant economic costs for the patient and the health service. It is therefore very important to better understand the local patterns of ocular injuries (through accurate data collection) and to develop appropriate prevention and management strategies.
