# Supplementary material for: Eye injuries: improving our practice
Source: Community Eye Health. 2015;28(91):41–3. (PMC4790157)
Supplement: Related texts

# Brief report: The BETTS classification system

**Published:** 2015

Although the eyewall technically also includes the choroid and retina, only the rigid structures (sclera and cornea) are taken into consideration in the Birmingham Eye Trauma Terminology System (BETTS). If an injury is atypical, or ‘mixed’, then clinicians can classify based on their best judgement or on the injury with the worst prognosis.

## Closed-globe injuries

- These are partial-thickness wounds of the eyewall.
- It is rare to find a **contusion** (caused by a blunt object) and a **lamellar laceration** (caused by a sharp object) together. In such cases best clinical judgment has to be used to describe it.
- With **lamellar lacerations**, a partial thickness wound is present and the clinician should specify whether it is corneal or scleral.
- **Contusions** involve bruising and swelling and may cause some structural changes in the shape of the globe, e.g angle recession.

## Open-globe injuries

- These are full-thickness wounds of the eyewall. The choroid and retina maybe intact, prolapsed or damaged.
- **Rupture** is caused by a blunt-object impact which produces an ‘inside-out’ force that ruptures the eyewall at its weakest point and can result in tissue herniation.
- A **laceration** is an ‘outside-in’ mechanism caused by a sharp object and resulting in a full-thickness wound at the impact site.
- A **penetrating injury** is a single laceration by a sharp object – there is only an entrance wound and no exit wound
- A **perforation** consists of two full-thickness lacerations caused by the same object (entrance and exit wounds).

**Figure:** Examining an eye injury patient using a slit lamp. CAMEROON

**Figure 1.** BETTS eye injury classification
